# Strategizing the Treatment Plan for Managing Comorbid Cervical Carcinoma With Coronary Artery Disease Using Coronary Artery Bypass Grafting and Definitive Oncotherapy

**DOI:** 10.7759/cureus.103139

**Published:** 2026-02-06

**Authors:** Rumi Kumari, Niraghatam Harshavardhan, Sneha Jain, Nighat Hussain, Nitin Kumar Kashyap

**Affiliations:** 1 Obstetrics and Gynecology, Pt. Jawahar Lal Nehru Memorial Medical College, Raipur, IND; 2 Pediatric Cardiothoracic Surgery, Sri Padmavathi Children's Heart Center, Tirupati, IND; 3 Cardiothoracic Surgery, All India Institute of Medical Sciences, Raipur, Raipur, IND; 4 Pathology and Laboratory Medicine, All India Institute of Medical Sciences, Raipur, Raipur, IND

**Keywords:** cervical carcinoma, chemoradiotherapy, cisplatin, coronary artery bypass grafting, coronary artery disease

## Abstract

Cancer and cardiovascular disease are the two most common reasons of mortality all over the world. It is common for both diseases to coexist because of the sharing of similar demographic features and risk factors such as obesity, old age, and smoking. Here, we present the case report of a patient with cervical carcinoma along with significant coronary artery disease, the purpose of which is to review planning such as considering percutaneous coronary intervention (PCI) vs coronary artery bypass grafting (CABG) or on-pump CABG vs off-pump CABG, the flowchart of management plans such as which disease needs to be addressed first, that is, whether it is the coronary artery disease or the carcinoma of the cervix, and the difficulties associated with the management of coexisting conditions.

## Introduction

The prevalence of cancer varies from 1.9% to 4.2% in cardiac patients, and 25% of patients with cancer are associated with cardiovascular illness [1-4]. Preoperative chemoradiotherapy for cancer might affect the left internal mammary artery (LIMA) harvesting, and several chemotherapy drugs are cardiotoxic as well [5]. Cardiac surgical decisions are influenced by various factors such as cancer prognosis, the location of the carcinoma, and the overall general condition of the patient. Myocardial revascularization improves the longevity of patients suffering from cancer coexisting with coronary artery disease, making subsequent oncotherapy safer [6,7]. The choice of modality for myocardial revascularization, i.e., percutaneous coronary intervention (PCI) vs coronary artery bypass grafting (CABG), in such patients plays a crucial role in determining the outcomes of post-treatment morbidity. The effect of cardiopulmonary bypass (CPB) on the spread of cancerous cells is not yet established [8,9]. Both humoral and cell-mediated immunity may be inhibited by the use of CPB [10]. Due to the presence of several influencing factors, different management strategies can be adopted.

## Case presentation

A 74-year-old woman with a prior diagnosis of carcinoma of the cervix, without radiologic evidence of distant metastasis, presented with complaints of chest pain and progressively worsening exertional dyspnoea of the New York Heart Association (NYHA) class III. The patient is a known case of well-controlled type 2 diabetes mellitus as well as hypertension for the past three years, along with recently diagnosed hypothyroidism.

**Figure 1:**
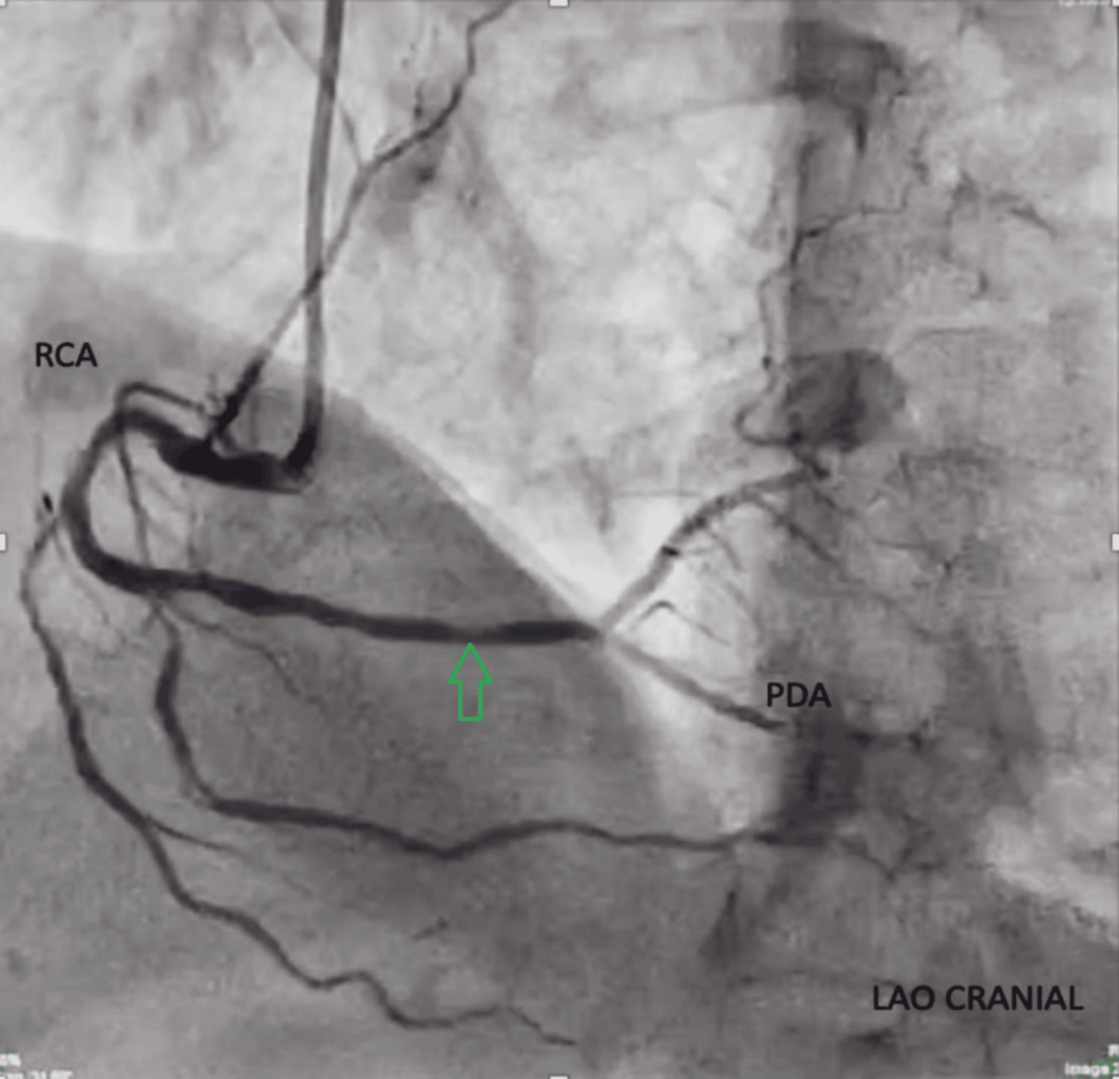
Right coronary angiogram in the left anterior oblique cranial view demonstrating the RCA with continuation into the PDA. The green arrow shows the diffuse plaque in the distal RCA and distal poor runoff RCA: right coronary artery; PDA: posterior descending artery; LAO: left anterior oblique

**Figure 2:**
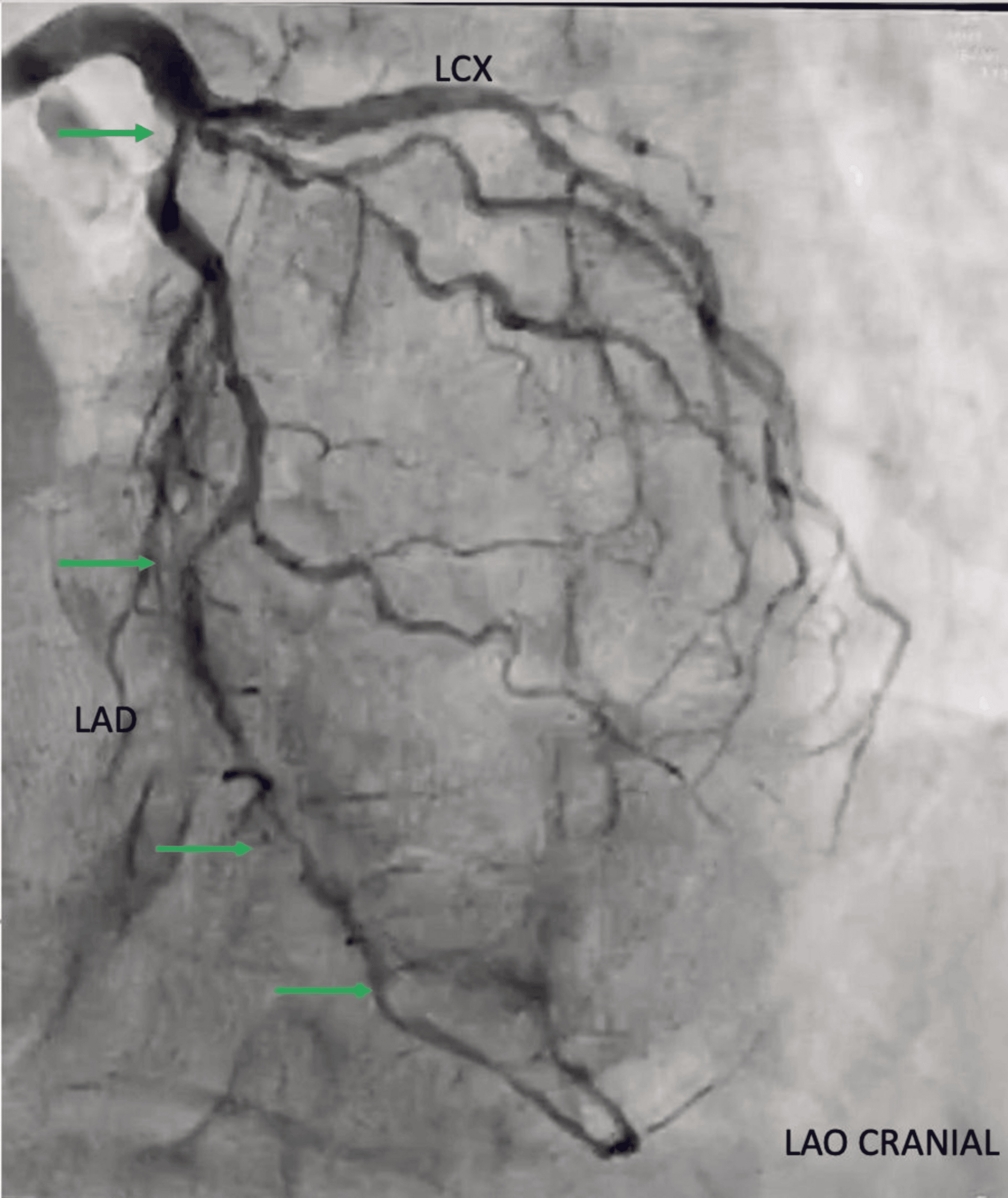
Coronary angiogram in the left anterior oblique cranial view showing the left main coronary artery bifurcating into the LAD artery and LCX. The green arrow shows the lesion in the ostio-proximal and mid-LAD followed by tandem lesions in LAD LAD: left anterior descending; LCX: left circumflex artery; LAO: left anterior oblique

At the time of admission, the patient was hemodynamically stable with a blood pressure of 160/94 mmHg, a pulse rate of 112 per minute, and an oxygen saturation of 96% on room air. Electrocardiography (ECG) was suggestive of sinus tachycardia with T-wave inversions in anterolateral chest leads V1-V4. All other systems examination and laboratory investigations were within normal limits. Transthoracic echocardiography demonstrated mild hypokinesia of the antero-apical myocardial territory and distal interventricular septum with an ejection fraction of 51%. Bilateral basal atelectatic bands without any pulmonary metastasis were noted on the computed tomography (CT) scan of the chest. Coronary angiography revealed a normal left main coronary artery. The left anterior descending (LAD) artery showed 70% ostial stenosis with diffuse disease in the mid-LAD and a tandem lesion in the distal LAD. The ramus intermedius (RI) artery demonstrated diffuse disease in the ostio-proximal segment. The left circumflex artery (LCX) has minor atherosclerotic plaques with 60% stenosis in the terminal obtuse marginal (OM). The right coronary artery (RCA) was ectatic and diffusely calcified throughout its course with a normal posterior descending artery (PDA) and posterior left ventricular branch (PLVB) (Figure 1 and Figure 2).

**Figure 3:**
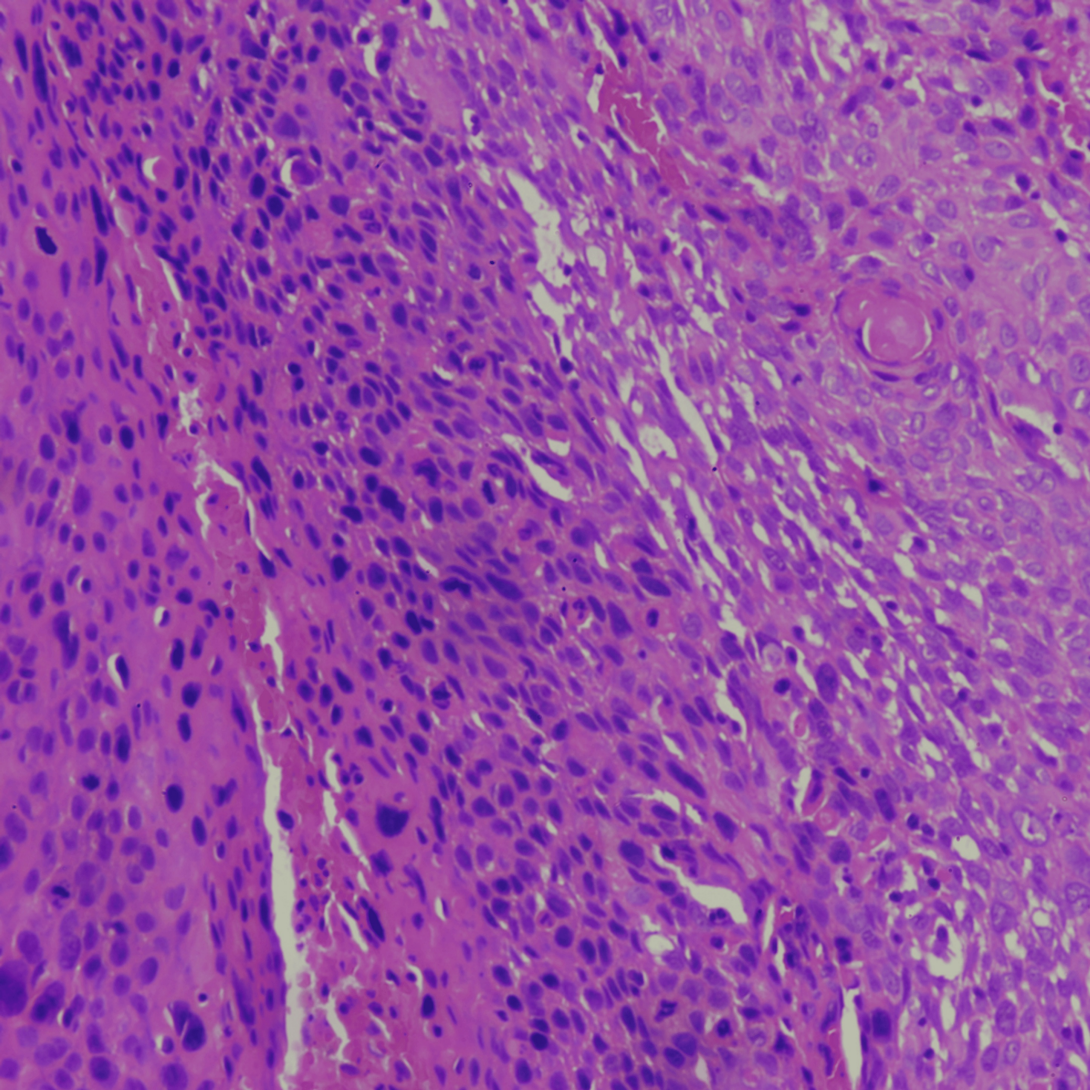
Histopathological examination of cervical malignancy demonstrating sheets of atypical squamous cells exhibiting intercellular bridges and well-formed keratin pearl (hematoxylin and eosin stain, ×10 magnification)

Histopathology examination of a biopsy from the cervix tissue revealed keratinizing squamous cell carcinoma (Figure 3).

Due to concurrent coronary artery disease with cervical malignancy, a multidisciplinary team (MDT) meeting was organized in which Oncology, Cardiac Sciences, Critical Care, and Gynecology teams participated, and all of them arrived at a consensus to perform CABG as the initial phase of the treatment plan. With regard to the management of coronary artery disease, CABG was preferred over PCI due to the characteristics of the lesions which were not amenable to PCI, that is, a high syntax score of >32 for all coronary lesions combined and the need for the usage of dual antiplatelet agents post-PCI which will increase the risk of bleeding in cases of active carcinoma of the cervix. The patient underwent off-pump CABG through a standard median sternotomy. Epicardial coronaries were dissected and were found to be calcified with poor flow on arteriotomy. Revascularization of the LAD was done using a reversed saphenous vein graft (rSVG) with a long vein patch technique. An rSVG conduit was used to graft the RI. Both venous conduits were anastomosed proximally to the ascending aorta separately. LIMA harvesting was not performed because LAD had tandem lesions with distal tubular stenosis pointing towards a poor distal runoff which will severely affect the patency of an arterial conduit. The distal RCA was heavily calcified, and it was decided not to perform grafting. This aspect did not influence the choice between on-pump and off-pump CABG. The patient recovered well and was discharged on postoperative day 8 and was planned for concurrent chemoradiation therapy, followed by intracavitary radiotherapy (ICRT) and cisplatin-based chemotherapy on an outpatient basis over a duration of 8-12 weeks in the postoperative period. Repeat follow-up radiology investigations after three months of chemoradiotherapy completion demonstrated no residual tumor. Furthermore, the patient is on scheduled outpatient department follow-up with no symptoms of angina and shortness of breath. Postoperative echocardiography was suggestive of left ventricular ejection fraction >50% with no wall motion abnormalities. The patient is able to manage her daily activities independently.

## Discussion

The surgical plan of patients presenting simultaneously with significant coronary artery disease along with malignancy is a complex scenario and requires a balanced approach in planning the sequence of treating these conditions. "Customized patient-centered strategy" based on tumor stage, cardiac risk profile, life expectancy, and patient preference is the key to successful outcomes in such patients keeping in mind few factors such as whether the resection of cancer is curative and does not have any metastasis, cardiac function is adequate with a left ventricular ejection fraction of at least 40%, and use of antiplatelet drugs such as aspirin 75 mg can be continued in the perioperative period [11]. Early oncologic treatment is necessary to prevent cancer progression, but untreated high-risk coronary artery disease may predispose patients to life-threatening cardiac events during chemoradiotherapy particularly with cardiotoxic agents such as cisplatin. Cisplatin-based regimens are highly effective in advanced cervical cancer patients but are associated with increased thromboembolic events, endothelial dysfunction, electrolyte derangements, and potential myocardial ischemia in patients with underlying coronary artery disease. Similarly, radiotherapy involving especially para-aortic or pelvic fields may cause microvascular injury and accelerated atherosclerosis. Early correction of coronary artery stenosis is therefore important to decrease the risk of acute coronary syndromes during the treatment of cancer [12]. Recent guidelines emphasize the importance of cardio-oncology collaboration in patients requiring both chemoradiotherapy and coronary revascularization [13]. The decision regarding the sequence of whether to treat coronary artery disease or cancer first depends on disease severity, urgency, cancer prognosis, and hemodynamic stability. In this particular patient, symptomatic multivessel disease with significant LAD stenosis represents the high-risk profile for peri-chemoradiotherapy myocardial ischemic events. MDT decision to prioritize CABG was made according to the current recommendations favoring the treatment of life-threatening cardiovascular conditions before initiating potentially cardiotoxic cancer therapy [14]. Off-pump CABG was planned to decrease inflammatory response, hemodynamic stress, and potential coagulopathy associated with on-pump surgery factors particularly relevant in older patients associated with malignancy [11]. Studies demonstrate that off-pump CABG reduces post-op morbidity and can be safely done in high-risk patients requiring early oncologic interventions. Uneventful postoperative recovery after CABG in this case led to the timely initiation of chemoradiotherapy, which is the treatment protocol for locally advanced cervical cancer. Restoring adequate coronary perfusion before cancer treatment likely reduces cardiac risk and improves tolerance to cancer therapy. Studies have shown that patients with coexisting CAD and malignancy who undergo coronary revascularization initially have improved short-term outcomes [15].

## Conclusions

With this case report, we highlight the importance of individualized multidisciplinary collaborative decision-making in patients with coexisting malignancy and significant cardiovascular disease for favorable short-term outcomes. Performing CABG prior to chemoradiotherapy enables the safe optimization of cardiac function and allows the timely commencement of cancer treatment, but this must be viewed with caution as it is a case report from a single center and it is difficult to draw significant conclusions from this. Oncological outcomes from this report need to be confirmed by longer follow-up which can be done in the future.
